# Solving the native structures of protein nanocrystals grown in bacteria by electron diffraction

**DOI:** 10.1063/4.0000930

**Published:** 2025-10-27

**Authors:** Marcus Gallagher-Jones

**Affiliations:** The Rosalind Franklin Institute, Harwell, Oxfordshire, United Kingdom

## Abstract

Bacillus thuringiensis (Bt) is a soil-dwelling bacteria that forms small crystalline inclusions within the confines of its cell wall as a response to starvation. These inclusions are composed exclusively of proteins with highly specific insecticidal activity, leading them to become the most widespread bio-pesticide worldwide. Unpicking the activity of these proteins requires a detailed understanding of both their structure and their arrangement within the crystal lattice. The small size of these inclusions makes them challenging for conventional structural biology as they are difficult to locate and their small number of unit cells produces much weaker Bragg scattering for a given flux. Electron microscopy can readily image nanocrystals and the larger scattering cross section of electrons relative to X-rays allows meaningful diffraction signals to be measured from far fewer crystalline repeats. In this work, we explore electron diffraction (specifically microED and serialED) as a tool for structure determination of Bt toxins in their native state. We demonstrate that electron diffraction methods are comparable and in some cases surpass other cutting-edge crystallographic methods such as serial femtosecond crystallography at Free electron lasers. The workflow presented should also apply to other sub-micron crystals that are highly beam-sensitive and difficult to study by other means.

